# Deep Learning-Based Identification of Intraocular Pressure-Associated Genes Influencing Trabecular Meshwork Cell Morphology

**DOI:** 10.1016/j.xops.2024.100504

**Published:** 2024-03-05

**Authors:** Connor J. Greatbatch, Qinyi Lu, Sandy Hung, Son N. Tran, Kristof Wing, Helena Liang, Xikun Han, Tiger Zhou, Owen M. Siggs, David A. Mackey, Guei-Sheung Liu, Anthony L. Cook, Joseph E. Powell, Jamie E. Craig, Stuart MacGregor, Alex W. Hewitt

**Affiliations:** 1 Menzies Institute for Medical Research, University of Tasmania, Hobart, Tasmania, Australia; 2 Centre for Eye Research Australia, University of Melbourne, Melbourne, Victoria, Australia; 3 Department of Information and Communication Technology, University of Tasmania, Hobart, Tasmania, Australia; 4 Statistical Genetics Laboratory, QIMR Berghofer Medical Research Institute, Brisbane, Australia; 5 Department of Ophthalmology, Flinders Medical Centre, Flinders University, Bedford Park, Australia; 6 Cellular Genomics Group, Garvan Institute of Medical Research, Sydney, New South Wales, Australia; 7 Faculty of Medicine and Health, School of Clinical Medicine, UNSW, Sydney, New South Wales, Australia; 8 Lions Eye Institute, Centre for Ophthalmology and Visual Science, University of Western Australia, Perth, Western Australia, Australia; 9 Wicking Dementia Research and Education Centre, University of Tasmania, Hobart, Tasmania, Australia; 10 Garvan-Weizmann Centre for Cellular Genomics, Garvan Institute of Medical Research, Sydney, New South Wales, Australia; 11 UNSW Cellular Genomics Futures Institute, UNSW, Sydney, New South Wales, Australia

**Keywords:** Glaucoma, Genetics, CRISPR, Transcriptomics, Morphological profiling

## Abstract

**Purpose:**

Genome-wide association studies have recently uncovered many loci associated with variation in intraocular pressure (IOP). Artificial intelligence (AI) can be used to interrogate the effect of specific genetic knockouts on the morphology of trabecular meshwork cells (TMCs) and thus, IOP regulation.

**Design:**

Experimental study.

**Subjects:**

Primary TMCs collected from human donors.

**Methods:**

Sixty-two genes at 55 loci associated with IOP variation were knocked out in primary TMC lines. All cells underwent high-throughput microscopy imaging after being stained with a 5-channel fluorescent cell staining protocol. A convolutional neural network was trained to distinguish between gene knockout and normal control cell images. The area under the receiver operator curve (AUC) metric was used to quantify morphological variation in gene knockouts to identify potential pathological perturbations.

**Main Outcome Measures:**

Degree of morphological variation as measured by deep learning algorithm accuracy of differentiation from normal controls.

**Results:**

Cells where *LTBP2* or *BCAS3* had been perturbed demonstrated the greatest morphological variation from normal TMCs (AUC 0.851, standard deviation [SD] 0.030; and AUC 0.845, SD 0.020, respectively). Of 7 multigene loci, 5 had statistically significant differences in AUC (*P* < 0.05) between genes, allowing for pathological gene prioritization. The mitochondrial channel most frequently showed the greatest degree of morphological variation (33.9% of cell lines).

**Conclusions:**

We demonstrate a robust method for functionally interrogating genome-wide association signals using high-throughput microscopy and AI. Genetic variations inducing marked morphological variation can be readily identified, allowing for the gene-based dissection of loci associated with complex traits.

**Financial Disclosure(s):**

Proprietary or commercial disclosure may be found in the Footnotes and Disclosures at the end of this article.

Primary open-angle glaucoma (POAG) is a blinding disease characterized by progressive degeneration of the optic nerve and retinal nerve fiber layer.^1^^,^^2^ Primary open-angle glaucoma is one of the leading causes of blindness globally.^3^ Whilst the precise pathophysiology of glaucoma is unknown, the most important modifiable risk factor is raised intraocular pressure (IOP).^1^^,^^4^ Raised IOP in POAG is primarily caused by dysfunctional aqueous humor drainage through the trabecular meshwork.^1^ Family heritage studies and genome-wide association studies (GWASs) have demonstrated a genetic contribution to trabecular meshwork dysfunction in POAG; however, the exact cellular and genetic processes involved remain unknown.^1^ Current treatments for POAG focus on reducing IOP by decreasing the production of aqueous humor or increasing outflow, with medications, or through the use of pressure-lowering surgery. However, there is currently no definitive cure for all patients with POAG.^5^ For novel pressure-lowering treatments to be developed, the pathophysiology of raised IOP in POAG must be understood and molecular pathways for this vision threatening disease uncovered.

Previous research has implicated a number of genes that contribute to POAG development and variation in IOP.^1^^,^^6^ Linkage analysis identified variants in the *MYOC* gene as being strongly associated with POAG.^7^, ^8^, ^9^ Disease-causing mutations in this gene have been shown to cause accumulation of a misfolded protein (myocilin), resulting in endoplasmic reticulum stress in trabecular meshwork cells (TMCs) and a subsequent rise in IOP.^6^ Genome-wide association studies have identified numerous genetic variants associated with raised IOP, many of which have also been associated with POAG.^10^^,^^11^ However, further investigation into these genetic variants is required to identify which individual genes may be affected by these variants and, thus, what cellular mechanisms may be involved. The ongoing development of artificial intelligence (AI) and deep learning tools such as convolutional neural networks (CNNs) provides a unique opportunity to investigate the genes of interest highlighted in GWAS and their effect on single cell morphology.

Deep learning is a rapidly advancing field of machine learning that relies on neural networks to learn abstract representations of data. A CNN is a specialized deep learning model designed to learn features of image data. In supervised learning, the original images are labeled, allowing CNNs to learn the correct representation for a given label. Given the effectiveness of CNNs at image classification,^12^ they have been extensively used in the analysis of cellular morphology, which is relevant in many domains of biology and medicine such as phenotype analysis,^13^^,^^14^ drug screening,^15^^,^^16^ and cell sorting.^17^^,^^18^

This study aimed to train a CNN to distinguish between primary TMCs that had specific genes from selected IOP-associated loci,^10^^,^^11^ knocked out using clustered regularly interspaced short palindromic repeats (CRISPR)/Cas, and control TMCs transfected with nontargeting guide RNAs. The accuracy, as measured by the area under the receiver operator curve (AUC) metric, was used to quantify variation in morphological profiles between target gene knockouts and control cells. This high-throughput approach uncovered genes at IOP loci, which, when perturbed, lead to marked variation in TMC morphology.

## Methods

### Cell Culture and Passaging

Primary TMCs were collected from a 58-year-old donor through the Lions Eye Donation service (Human Research Ethics Committee of the Royal Victorian Eye and Ear Hospital - reference number 13-1151H). Cells were cultured in Dulbecco’s Minimal Essential Medium (Gibco, 11965118) with 10% fetal bovine serum (Gibco, 16000044) and 0.5% antibiotic-antimycotic (Gibco, 15240-062) (herein referred to as “culture medium”) at 37°C with 5% CO_2_. Cells were passaged by removing the culture medium and washing twice with phosphate buffered saline (Gibco, 14190144). Trypsin 0.25% diluted in phosphate buffered saline (Gibco, 25200056) was then added and the cells were incubated for 3 minutes at 37°C with 5% CO_2_. The trypsin was deactivated with cell culture medium and cells were then aspirated into tubes and centrifuged at 1000 rpm for 5 minutes. The supernatant was aspirated and the cell pellet was resuspended in culture medium before being plated at the desired ratio for ongoing culture. All TMCs were cultured in tissue-culture treated polystyrene plates (Corning, 3516, 3524). Prior to use, our primary TMCs were characterized as previously described.^19^ In brief, we verified phagocytosis and expression of CAV1 and TIMP3 by immunostaining, as well as *MYOC* induction by dexamethasone exposure (Fig S1). Cell lines were tested for mycoplasma on an alternate weeks using the PCR Mycoplasma Test Kit (PromoKine, PK-CA91-1096). The primary TMCs were regarded as passage zero and were seeded at passage 3 before undergoing respective cell painting immunohistochemistry or RNA-sequencing protocols at passage 4.

### Cell Transfection and CRISPR Gene Knockout

A total of 67 TMC lines were generated using a library of 124 targeting single guide RNAs (sgRNAs) (2 for each target gene), together with 10 nontargeting sgRNAs as negative controls. Single guide RNAs were designed using GUIDES^20^ and are displayed in Table S1. Following synthesis, sgRNAs were cloned into a novel construct that had previously been developed for the pooled single-cell RNA sequence profiling of primary cells (CROPseq-Guide-pEFS-SpCas9-p2a-puro; Addgene: #99248).^21^ The lentivirus was then packaged by transfecting human embryonic kidney 293FT cells with pCMV delta 8.91, pMDG, and the recombinant plasmid via lipofectamine 2000. Lentivirus was chosen as the optimal viral vector due to its large size of ∼8.5 kB allowing sgRNA, Cas9, and puromycin resistance genes to be packaged into 1 viral vector.^22^

P1 primary TMCs were transfected with 50 μl of lentiviral plasmid to give a multiplicity of infection of approximately 3, and each CRISPR/Cas9/sgRNA/puromycin plasmid in an arrayed format. Individually cloned CRISPR/Cas9/sgRNA/puromycin plasmids were separately added to 450 μl of TMCs in culture mixed with 1:100 lentiblast (OZ Bioscience, LB01500) in 24 well plates. Each well was seeded with approximately 3.0 × 10^4^ cells. Cell cultures were incubated for 3 days before 1 μg/ml puromycin selection occurred over 4 days. Transfected TMCs underwent standard cell passaging and were then resuspended in 100 μl to 500 μl Dulbecco’s Minimal Essential Medium depending on initial cell density. Initial cell density was qualitatively checked with brightfield microscopy before seeding. The predicted on-target editing efficiency for each sgRNA was generated for each sgRNA (Table S1). The mRNA expression of each gene knockout can be quantified from RNA sequencing data; however, while CRISPR introduces indels into the targeted sequence, the transcription of mRNA for each target gene still occurs. To investigate the efficacy of these CRISPR-constructs we compared the targeted gene transcript in each knockout line to that of the nontargeting control cells. Overall, 25 of the target cells had lower transcript counts compared with the controls at the Bonferroni corrected level (*P* < 0.0008; Fig S2), which is reassuring given that the transcripts would be transcribed though susceptible to nonsense mediated decay.

### Cell Painting and Imaging Protocols

**Figure 1:**
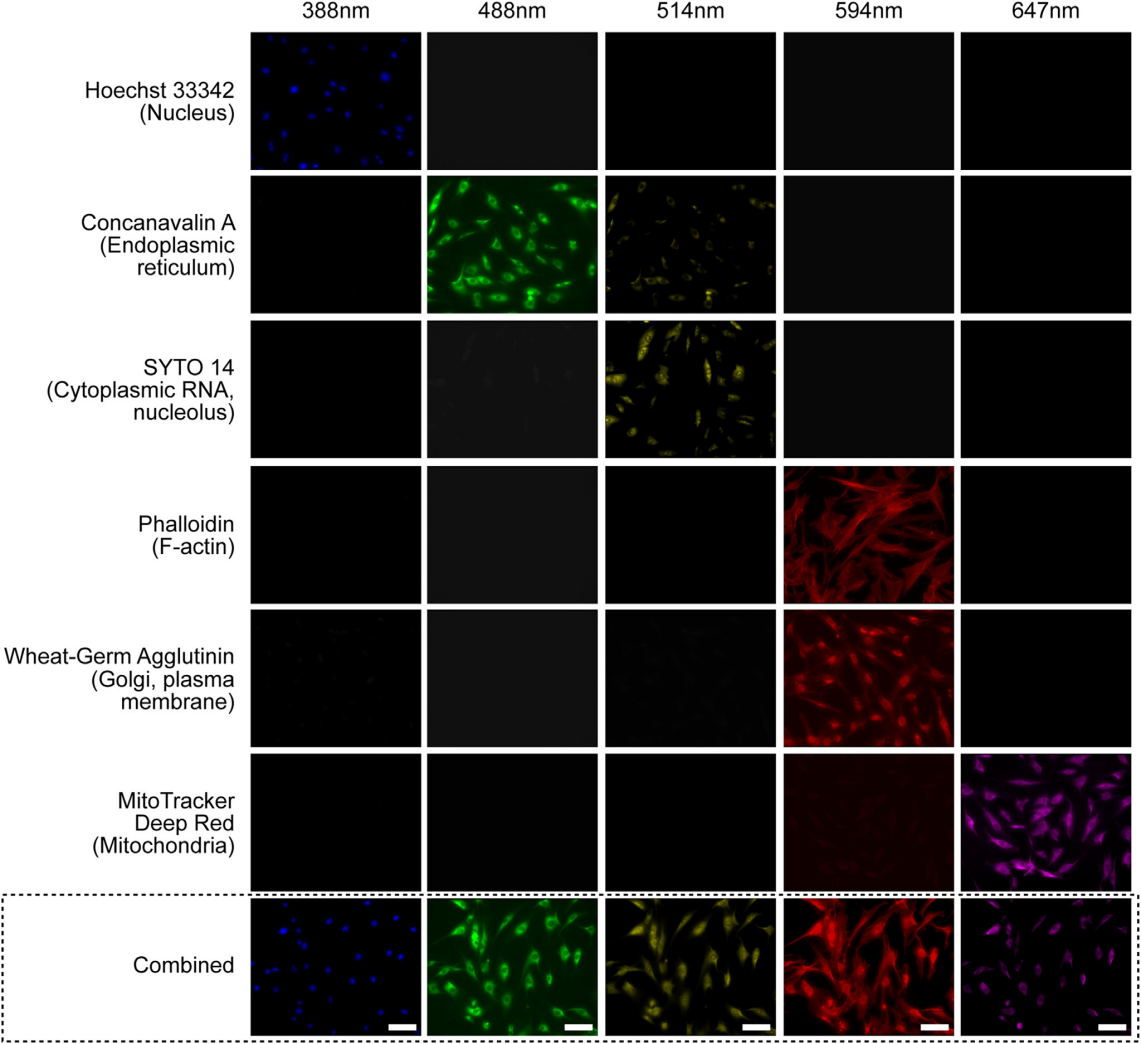
Cell Painting of trabecular meshwork cells (TMCs). Example images of TMCs stained with the Cell Painting protocol in which 6 fluorophores are imaged over 5 channels to identify 8 distinct intracellular organelles for morphological profiling. Each row shows different cells stained with the single fluorophore, or 1 group with all fluorophores combined (bottom row); columns indicate excitation wavelengths. Single channel testing shows minimal overlap across channels except for the phalloidin and wheat germ agglutinin stains which are analyzed together. This ensures that only a single stain will fluoresce when exposed to a particular wavelength of light. This figure shows whether a single stain would contaminate other emission channels and whether the signal of the light emission channel was dominated by the dyes we selected. Scale bar 50 μm.

Cells were seeded at random in triplicates across 96 well plates at a density of 4.0 × 10^3^ cells per well using a Beckman Coulter MoFlo Astrios EQ fluorescence-activated cell sorter to ensure an equal distribution of cells. The Cell Painting protocol as described by Bray et al^23^ was then followed. Briefly, TMCs were incubated in culture medium containing 500 nM Mitotracker (Invitrogen, M22436) and 30 μg/ml wheat germ agglutinin Alexa594 conjugate (Invitrogen, W11262) for 30 minutes at 37°C. Then TMCs were fixed with 4% paraformaldehyde at room temperature for 20 minutes and washed with 150 μl of Hanks' balanced salt solution (HBSS) (Gibco, 14025134). Next, TMCs were permeabilized with 0.1% solution of Triton X-100 (Sigma, T8787) for 20 minutes and washed with 150 μl HBSS twice. Lastly, TMCs were incubated with HBSS staining solution containing 1% bovine serum albumin (Merck, A8806), 50 μg/ml ConcanavalinA (Invitrogen, C11252), 3 μM Syto14 (Invitrogen, S7576), 5 μg/ml Hoechst (Invitrogen, H3570), and 1 unit/ml Phalloidin (Invitrogen, A12381) for 30 minutes at room temperature. Trabecular meshwork cells were washed 3 times with HBSS without final aspiration and then sealed with parafilm. All 96 well plates were kept at 4°C in the dark before imaging. Then the TMCs were imaged with high content microscopy taken at 20× magnification across 5 fluorescent channels on a Zeiss CellDiscoverer7 as outlined in Table 1. Images were autofocused using the definite focus strategy (a set focus point for each image) at 25 sites per well as shown in Figure 1.

**Table 1:** Cell Painting Reagents, Fluorescent Channels, and Associated Cellular Organelles

Cell Painting Reagent	Fluorescent Channel	Excitation Filter (nm)	Emission Filter (nm)	Organelles
Hoechst 33342	DAPI	387/11	417–477	Nucleus
Concanavalin A/Alexa Fluor 488 conjugate	EGFP	472/30	503–538	Endoplasmic reticulum
SYTO 14 Green Fluorescent Nucleic Acid stain	AF514	531/40	573–613	Cytoplasmic RNA, nucleolus
Phalloidin/AlexaFluor 568 WGA/AlexaFluor 555 conjugate	AF594	581/609 (phalloidin) 590/617 (WGA)	622–662	F-actin, golgi complex, cell membrane
MitoTracker deep red	AF647	628/40	672–712	Mitochondria

WGA = wheat germ agglutinin.

The Cell Painting protocol was designed to allow a maximum number of cellular organelles to be visualized with minimal overlap of fluorescent channels.

### Image Preprocessing and Quality Control

All images were separated into multiple single-cell images using the “Save Cropped Objects” function in CellProfiler (version 3.1.9, Broad Institute, Massachusetts Institute of Technology).^24^^,^^25^ This was undertaken to ensure that single-cell morphology was the only feature of the image, and classification was not influenced by overall cell confluency. An image quality filter was then applied using CellProfiler, which flagged any low-quality images that may contain artefacts or were inadequate for analysis, and these were subsequently removed. CellProfiler analysis data was used to calculate Spearman's rank correlation of individual cells for all cell lines. Noncorrelated cells from each line were then removed by setting a Spearman correlation cutoff value of 0.15 to reduce well-to-well and batch-to-batch variation.

### CNN Architecture, Training, and Evaluation

The CNN architecture is outlined in Table S2 and accessible via GitHub. We sought to randomly select 3000 cell images from control and gene knockout groups to allocate into training (80%), validation (10%), and testing (10%) sets. A separate CNN was trained for each fluorescent channel of each gene across 5 replicates (each with a different random seed to create individual datasets). Training was conducted for 100 epochs, with the model being saved at each epoch. An Adam optimizer was used with a learning rate of 0.0001. For evaluation, the best performing model of the 100 epochs as per the loss function was selected and evaluated on the test set. Testing was performed by training a network which sought to distinguish control images from gene knockouts and the AUC metric was used to quantify CNN performance and thus, the degree of morphological variation induced by genetic variations. The highest performing models were all selected prior to reaching 100 epochs where model overfitting began to reduce model accuracy.

## Results

### Image Filtering and Data Split

**Figure 2:**
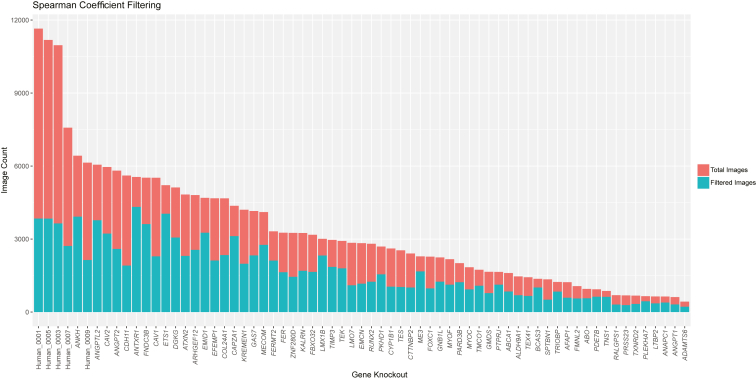
Total number of images for each arrayed cell line following Spearman correlation filtering. Images were removed from the dataset if the Spearman correlation was > 0.15 in order to improve the quality of the dataset and reduce the effect of well-to-well and batch-to-batch variation. Ultimately, the percentage of cells removed ranged from 67% (control line 1) to 22% (*ANTXR1*).

Filtering using CellProfiler and by Spearman correlation reduced the total dataset size from 225 095 images per channel to 114 830 images per channel, yielding a total of 574 150 images for analysis. The proportion of images removed via Spearman filtering varied across groups from 22.1% (*ANTXR1*) to 70.0% (nontargeting group 1). The 5 nontargeting control lines had the greatest proportion of images removed via Spearman filtering as shown in Figure 2. The total number of cell images after filtering ranged from 221 (*ADAMTS6*) to 4323 (*ANTXR1*). This intergroup variability was balanced during training with image rotation data augmentation (0, 90, 180, 270, with or without horizontal mirroring) to reach 3000 images per group. When 3000 were not obtained using data augmentation, the control group numbers were reduced to match and maintain a 50:50 balanced split between knockout and control images. This occurred in only 6 knockout cell lines (*ADAMTS6*, *PRSS23*, *RALGPS1*, *ANGPT1*, *TXNRD2*, and *LTBP2*) which had 1768, 2296, 2488, 2520, 2688, and 2872 images, respectively. A random selection of nontargeting control images was then selected to produce a balanced dataset of gene knockout and nontargeting control images. The same nontargeting images were chosen for each knockout comparison. The dataset was split into training (80%), validation (10%), and testing (10%) sets.

### Overall Morphological Variation Induced by Genetic Knockouts

**Figure 3:**
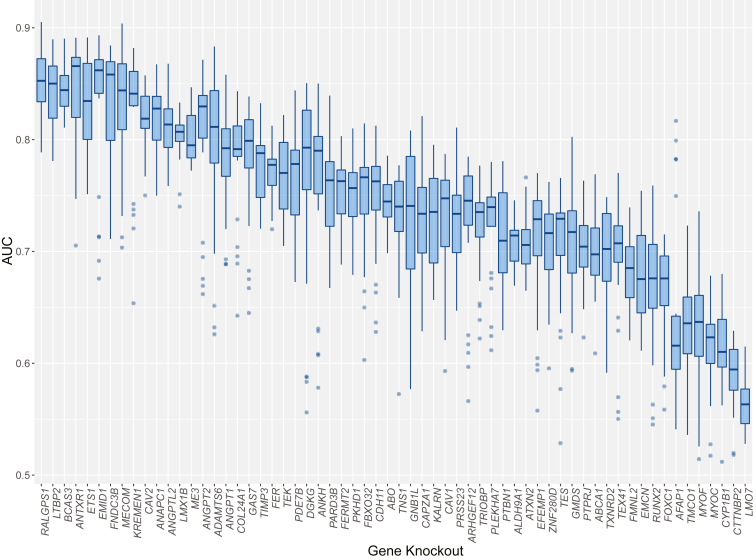
Mean convolutional neural network (CNN) area under the receiver operator curve (AUC) scores for each gene knockout cell line. The mean AUC score when training a CNN to distinguish between gene knockout cell lines and nontargeting control cell lines. A higher AUC indicates a more distinct morphological variation induced by a particular gene knockout. The gene knockouts are in decreasing order of mean AUC across all organelles. The bars represent the median AUC with upper and lower quartile boxes. Outliers are displayed as single dots.

The AUC metric was used to assess the ability of the CNN to distinguish genetic knockout lines from nontargeting control lines, thereby providing a quantifiable value of morphological variation induced by gene knockouts. The mean AUC of 5 replicates across 5 channels was calculated to produce an overall AUC for each target gene. Knockout of *RALGPS1* produced the most morphologically distinct TMCs (AUC 0.851, standard deviation [SD] 0.030); however, the *RALGPS1* cell line was not significantly knocked down by the CRISPR system (*P* = 0.605) as seen in Fig S2. The second most morphologically distinct was *LTBP2* (AUC 0.846, SD 0.029), followed by *BCAS3* (AUC 0.845, SD 0.020). Both *LTBP2* and *BCAS3* had statistically significant gene knockout efficacy (*P* = 2.22 × 10^−16^ and 2.29 × 10^−3^). The overall AUCs ranged from 0.564 (*LMO7*) to the most distinguishable at 0.851 (*RALGPS1*) as displayed in Figure 3.

### Morphological Variation Induced in Individual Organelles

**Figure 4:**
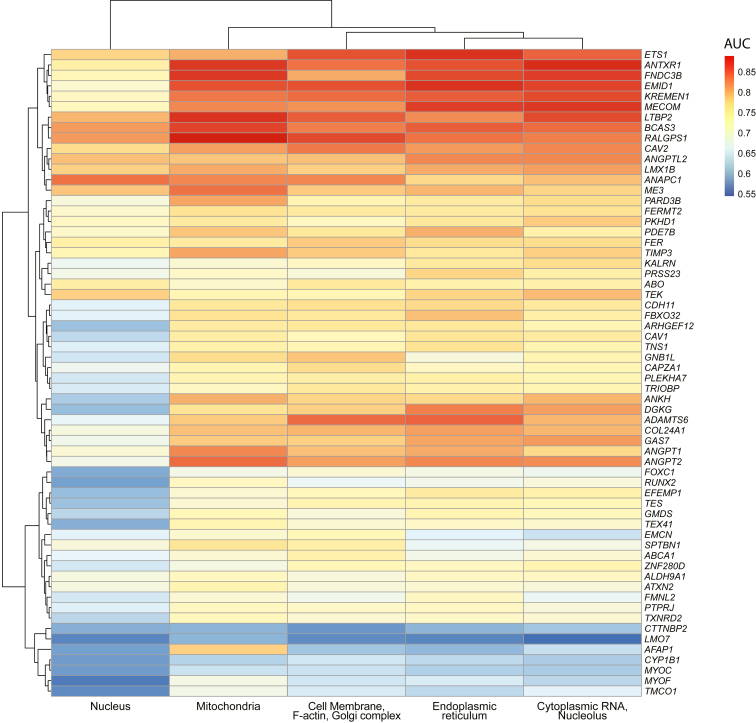
Gene knockout cell line area under the receiver operator curve (AUC) for each organelle. Heatmap of the morphological variation (AUC) across individual fluorescent channels for each gene knockout. Red shading indicates a higher degree of morphological variation as indicated by a higher AUC.

Twenty-one (33.9%) gene knockout groups had greater morphological distinction in the mitochondrial channel (mean AUC 0.760 of all cell lines, SD 0.070) compared with other organelles, illustrating that mitochondrial variation occurs in a large proportion of the gene knockouts. The relative AUC of each gene across all organelles is shown in Figure 4. Endoplasmic reticulum showed the next greatest morphological variation evident in 16 (25.8%) of the gene knockout lines (mean AUC 0.756, SD 0.079). The F-actin/cell membrane/Golgi body channel showed the highest morphological variation in 13 (20.9%) gene knockout lines (mean AUC 0.751, SD 0.073), followed by 11 (17.7%) knockout lines in the cytoplasmic RNA/nucleolus channels (mean AUC 0.753, SD 0.078). Finally, only the *ANAPC1* knockout showed morphological variation most in the nucleus (mean AUC 0.677, SD 0.079).

### Gene Prioritization

Finally, we used the trained CNN AUC metrics to investigate TMC morphological variation for genes at multigene loci.^10^^,^^11^ Table 2 displays the AUC (knockout of target gene compared to nontargeting control) for 15 genes at overlapping IOP associated loci. These analyses prioritized 4 gene knockouts (*ALDH9A1*, *CAV2*, *ME3*, and *RALGPS1*) at these loci, which resulted in greater morphological variation than knockout of their neighboring gene counterparts. However, when considering gene knockdown efficacy, of these 4 targets only *ALDH9A1* and *CAV2* had statistically significant changes in respective gene expression (*P* = 3.47 ×10^−2^ and 2.22 ×10^−16^, respectively). Knockout of genes at 2 multigene loci (*EMID1-KREMEN1* and *GNB1L-TXNRD2*) generated TMCs that were morphologically similar and thus could not be resolved. *KREMEN1* had a statistically significant knockdown effect (*P* = 1.84 ×10^−11^) while the remaining 3 had no significant change in gene expression, which may explain why these multigene loci could not be resolved.

**Table 2:** Comparison of Convolutional Neural Networks to Morphologically Distinguish Trabecular Meshwork Cells With Knockout of Genes at Overlapping IOP-Associated Loci^10^

Top IOP GWAS SNP	Overlapping Genes (Mean AUC)	*P* Value
rs7518099	*ALDH9A1* (AUC 0.709, SD 1.93e-02) *TMCO1* (AUC 0.634, SD 4.76e-02)	7.78 × 10^−05^
rs11795066	*RALGPS1* (AUC 0.851, SD 3.05e-02) *ANGPTL2* (AUC 0.811, SD 2.50e-02)	4.12 × 10^−04^
rs6478746	*LMX1B* (AUC 0.803, SD 2.03e-02) *RALGPS1* (AUC 0.851, SD 3.12e-02)	5.5 × 10^−06^
rs10281637 rs55892100	*CAV1* (AUC 0.726, SD 5.53e-02) *CAV2* (AUC 0.817, SD 2.71e-02) *TES* (AUC 0.704, SD 5.79e-02)	4.49 × 10^−01^ (*CAV1* vs. *TES*) 3.00 × 10^−03^ (*CAV2* vs. *TES*) 4.00 × 10^−03^ (*CAV1* vs. *CAV2*)
rs9608740	*EMID1* (AUC 0.834, SD 6.50e-02) *KREMEN1* (AUC 0.824, SD 5.70e-02)	5.73 × 10^−01^
rs8141433	*GNB1L* (AUC 0.729, SD 5.97e-02) *TXNRD2* (AUC 0.695, SD 4.47e-02)	3.75 × 10^−01^
rs746491	*ME3* (AUC 0.803, SD 2.45e-02) *PRSS23* (AUC 0.725, SD 4.25e-02)	3.47 × 10^−04^

AUC = area under the receiver operator curve; GWAS = genome-wide association studies; IOP = intraocular pressure; SD = standard deviation.

The mean AUC across all fluorescent channels of target knockouts vs. nontargeting control cells was compared for genes at the same locus. A higher AUC indicates a larger degree of morphological variation compared with normal control cells. This allows for prioritization of overlapping genes at given loci.

## Discussion

There has been a shift in recent years towards using high-throughput profiling to undertake large-scale studies investigating the cellular basis of disease. This shift has been accelerated by advancements in computational technology and AI as a method of rapidly analyzing large, complex datasets. In this study, we utilized a CNN to perform a high-throughput morphological analysis of genetic variations associated with IOP variation in primary human TMCs. By training the CNN to distinguish gene knockout cells from healthy control cells, we could use the AUC as a metric to quantify differences in cellular morphology induced by various genetic variations. Therefore, the AUC can be used to identify which variations invoke a greater degree of morphological change and thus, which are more likely to be involved in IOP dysregulation and the pathogenesis of POAG.

This study highlights the complex genetic basis of POAG, and has clinical relevance in the development of new therapeutics to treat this vision-threatening disease. If the precise pathophysiology of POAG can be understood at the cellular level, new drug targets may be uncovered. Further, the characterization of gene-based perturbations in TMCs is an important first step in the high-throughput screening of TMC modulators. Herein, we have described an AI framework for the large-scale profiling of TMCs.

Of the genes known to cause primary congenital glaucoma or anterior segment dysgenesis, *LTBP2* and *TEK* showed marked differentiation from normal control morphology. The *LTBP2* knockout cell line was readily distinguished from normal control TMCs (AUC 0.846) with the greatest degree of difference occurring in mitochondrial morphology, indicating that *LTBP2* may play a role in mitochondrial function. *LTBP2* encodes for latent transforming growth factor beta binding protein 2, which is an extracellular matrix protein associated with fibrillin-1 containing microfibrils and is hypothesized to modulate extracellular matrix production.^26^ Variations in *LTBP2* have been previously associated with primary congenital glaucoma, microspherophakia, megalocornea, and Weill-Marchesani syndrome.^26^, ^27^, ^28^, ^29^ A previous study has identified that *LTBP2* knockout may contribute to the development of POAG via dysregulation of the extracellular matrix, a crucial component of the trabecular meshwork.^30^ Studies looking at dilated cardiomyopathy and right ventricular failure have also implicated *LTBP2* function in fibrosis regulation which may indicate a role in the pathogenesis of trabecular meshwork dysfunction.^31^^,^^32^ Interestingly, although *LTBP2* encodes for an extracellular protein, we demonstrated a distinct mitochondrial morphology in *LTBP2* knockout cell lines for which we speculated that *LTBP2* may lead to oxidative stress on mitochondria either directly or via changes in gene expression involved in TGFβ and bone morphogenetic protein signaling pathway that may affect mitochondrial function.

The *TEK* knockout cell line also showed significant differentiation (AUC 0.768) most prominent in the cytoplasmic RNA and nucleolus channel. Variations in *TEK* have been associated with raised IOP and congenital glaucoma primarily due to disruption of Schlemm’s canal, indicating a potential interaction with *ANGPT1* in the development of glaucoma.^33^, ^34^, ^35^, ^36^ However, it must be noted that the knockdown efficacy of *TEK* in this study was insignificant (*P* = 0.858), indicating that the learned morphological variation may be due to a confounding factor learned by the deep learning system. *MYOC*, *CYP1B1*, *GMDS*, and *FOXC1* knockouts resulted in only mild differentiation from control TMC morphology (AUCs of 0.615, 0.612, 0.704, and 0.665, respectively) despite an association with glaucoma and anterior segment dysgenesis;^7^^,^^37^, ^38^, ^39^, ^40^ however, these genes were seen to have low knockout efficiency which may explain the limited morphological change. Additionally, these gene knockouts may not invoke significant morphological variation as they are primarily involved with trabecular meshwork development rather than their homeostatic maintenance.^41^ Furthermore, some gene mutations associated with congenital glaucoma are gain-of-function mutations, and may not show significant change when knocked out. Another reason for not seeing change in cellular morphology is that these genes may primarily act extracellularly, such as *MYOC*, which has been shown to demonstrate accumulation of extracellular products in specific mutations.^42^

Overall, the mitochondrial channel most frequently displayed the greatest degree of differentiation (33.9% of all cell lines). This supports previous work, where TMCs in POAG have been shown to demonstrate mitochondrial dysfunction resulting in sensitivity to calcium stress.^43^ The endoplasmic reticulum channel also showed the most morphological variation in a large proportion of cell lines (25.8%), which is in keeping with many studies that have highlighted a link between glaucoma and endoplasmic reticulum stress.^44^, ^45^, ^46^

This work introduced a novel method for prioritizing genes at overlapping loci identified in GWAS using CNN analysis.^10^^,^^11^ The results show that *ALDH9A1* and *CAV2* show statistically greater differentiation from control cells than the respectively associated gene at the same locus. Studies have previously associated POAG with genetic variants at the intergenomic region of *TMCO1* and *ALDH9A1*.^47^, ^48^, ^49^ The results of this study point toward *ALDH9A1* being the implicated gene in POAG due to inducing a greater degree of morphological change compared with *TMCO1* (*P* = 7.78 × 10^−05^). The mitochondrial channel in *ALDH9A1* displayed the greatest degree of differentiation, highlighting the potential role of mitochondrial dysfunction in *ALDH9A1* interruption in POAG. This is supported by the role of *ALDH9A1* in carnitine synthesis, which takes place in the mitochondrial matrix.^50^ There have also been numerous studies illustrating an association between POAG and variations at the intergenomic region of *CAV1* and *CAV2*.^51^, ^52^, ^53^, ^54^ This analysis prioritized *CAV2* as a potential causative gene, with a higher degree of morphological change from control cells than *CAV1* (*P* = 4.00 ×10^−03^). The *CAV2* knockout cell line displayed the most prominent changes in the F-actin, Golgi complex, and cell membrane fluorescent channel. Supporting this, previous studies have highlighted the interaction between *CAV2* and the Golgi complex.^55^, ^56^, ^57^ The remaining genes at overlapping loci (*EMID1* vs. *KREMEN1* and *GNB1L* vs. *TXNRD2*) showed no statistically significant differences in morphology as well as limited gene knockdown efficiency. They will require further investigation to prioritize which of these may be the causative gene.

A further application of AI-based analysis of single cell morphology is to predict gene expression as demonstrated in prior studies. For example, Chlis et al^58^ developed a machine learning model to predict gene expression of human mononuclear blood cells and mouse myeloid progenitor cells based on cellular morphology. Our study further highlights the complex interaction between cell morphology and gene expression and the opportunity that AI poses as a means of analyzing the large amounts of data produced. Further investigation into this field could uncover the genetic drivers behind pathological changes in morphology that drive disease processes and allow for identification of novel therapeutic targets.^58^^,^^59^

One of the main limitations of this study lies in the intrinsic difficulty in interpreting the decision-making process of CNNs. This means it can be difficult to establish if morphological features learned by the CNN are truly pathological or simply due to systematic bias. For example, if wells had lower cell density, the cells may grow to a larger size, and as such cell size may inadvertently influence the decision-making of the CNN. Certain gene knockouts may invoke cell death, which may account for lower cell numbers in particular cell lines as illustrated in Figure 2. A potential solution to this is to utilize attention-based CNN models which highlight areas of interest within the image used for decision-making.^60^ This may reveal which cell features are responsible for morphological variation and if features such as cell size or cell density are contributing. In addition, alternative functional systems, such as animal models, could be used to validate our findings. A further limitation of this study is that TMC cell lines were generated from a single donor which may reduce the generalizability of these results to the general population. Of course, carrying out such a study with larger donor numbers would provide a more robust dataset and increase generalizability. As well as this, it was noted that a number of cell lines had limited gene knockout efficacy as demonstrated by insignificant variations in gene expression (Fig S2). Nevertheless, this study does provide the foundations for similar larger scale studies to follow with greater donor numbers. Finally, given that we applied the Cell Painting protocol as described by Bray et al,^23^ which combines wheat germ agglutinin and phalloidin into the same fluorescent channel (Fig 1), it was challenging for us to distinguish actin cytoskeleton from Golgi apparatus and cell membrane features. Future studies may be able to expand on the use of Cell Painting by utilizing phalloidin alone in a separate assay to assess cytoskeletal changes that have been proposed to contribute to POAG.

In summary, this study used a powerful approach to quantify morphological change induced by genetic variations associated with POAG. *RALGPS1* produced the greatest morphological variation. In addition, we could prioritize genes at overlapping loci identified to have an association with IOP. However, there are some limitations due to the difficulty in removing systematic bias from the methodology. This bias may result in the CNN learning features that are not directly associated with IOP physiology. This study highlights a new avenue for utilizing CNNs trained on single-cell morphology to further interpret the results of GWASs.
