# Supplementary material for: Deep Learning-Based Identification of Intraocular Pressure-Associated Genes Influencing Trabecular Meshwork Cell Morphology
Source: Ophthalmol Sci. 2024 Mar 5;4(4):100504. doi: 10.1016/j.xops.2024.100504 (PMC11046128; doi:10.1016/j.xops.2024.100504)
Supplement: Appendix [file mmc1.pdf]

Code availability

The Python functions utilised for data preparation, CNN training and evaluation are available on GitHub: https://github.com/ConnorG1/TMC_CNN

Data availability

Data is available at the European Bioimage Institute Bioimage Archive: Accession S-BSST841
