# Supplementary material for: Deep Learning-Based Identification of Intraocular Pressure-Associated Genes Influencing Trabecular Meshwork Cell Morphology
Source: Ophthalmol Sci. 2024 Mar 5;4(4):100504. doi: 10.1016/j.xops.2024.100504 (PMC11046128; doi:10.1016/j.xops.2024.100504)

SUPPLEMENTARY DETAILS

Supplementary Figure 1: Characterization of primary TMCs. A) Immunostaining of CAV1 (cat.3238S, Cell Signalling); B) Immunostaining of TIMP3 (cat.ab39184, Abcam); C) Phagocytosis assay using pHrodo bioparticles (Red) (cat.A10010, ThermoFisher Scientific); D) Immunostaining of MYOC (PAH586Hu01, Cloud-Clone Corp.) without dexamethasone treatment; E) Immunostaining of myocilin (PAH586Hu01, Cloud-Clone Corp.) following dexamethasone exposure; F) Relative expression of MYOC with or without exposure to dexamethasone (normalized to EEF2 expression). Significant results are defined as *$p<0.05$; **$p<0.01$; ***$p<0.001$, and ****$p<0.0001$. Scale bar: 20 μm.

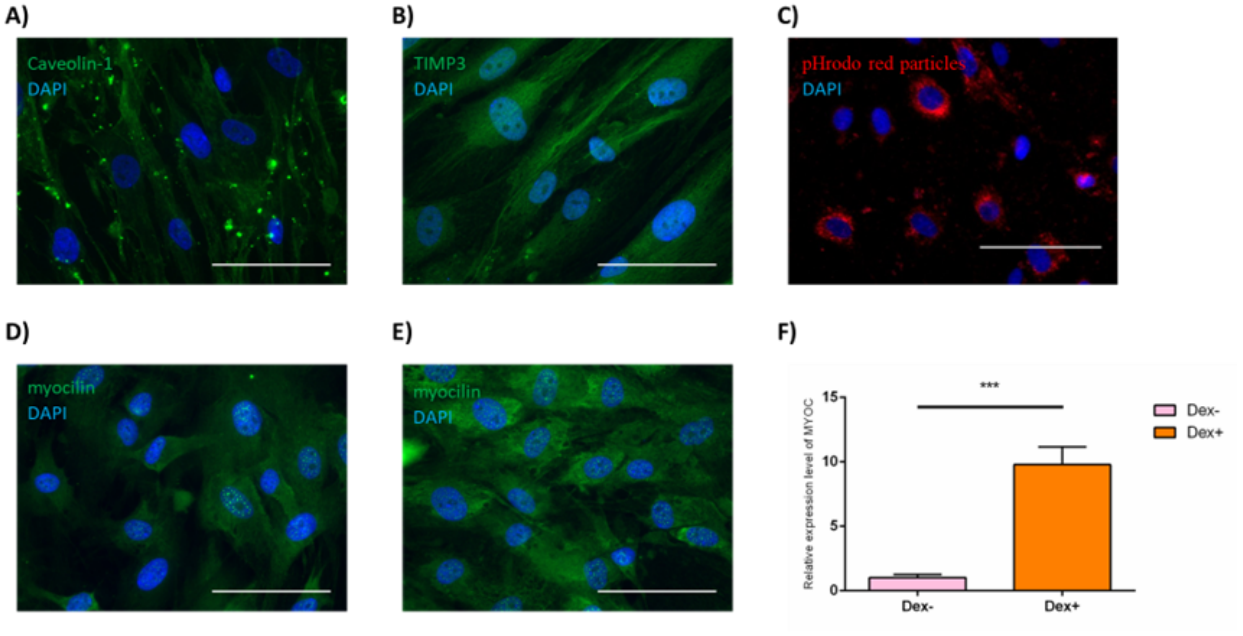

Supplement: Fig S1 [file mmc2.pdf]
