# Supplementary material for: Deep Learning-Based Identification of Intraocular Pressure-Associated Genes Influencing Trabecular Meshwork Cell Morphology
Source: Ophthalmol Sci. 2024 Mar 5;4(4):100504. doi: 10.1016/j.xops.2024.100504 (PMC11046128; doi:10.1016/j.xops.2024.100504)

Supplementary Figure 2: Comparison of gene expression levels between each target knockout cell line and non-targeting control cells. ABO and TEX41 were found not to be expressed in our TMCs. P-values are displayed from the Student t-test.

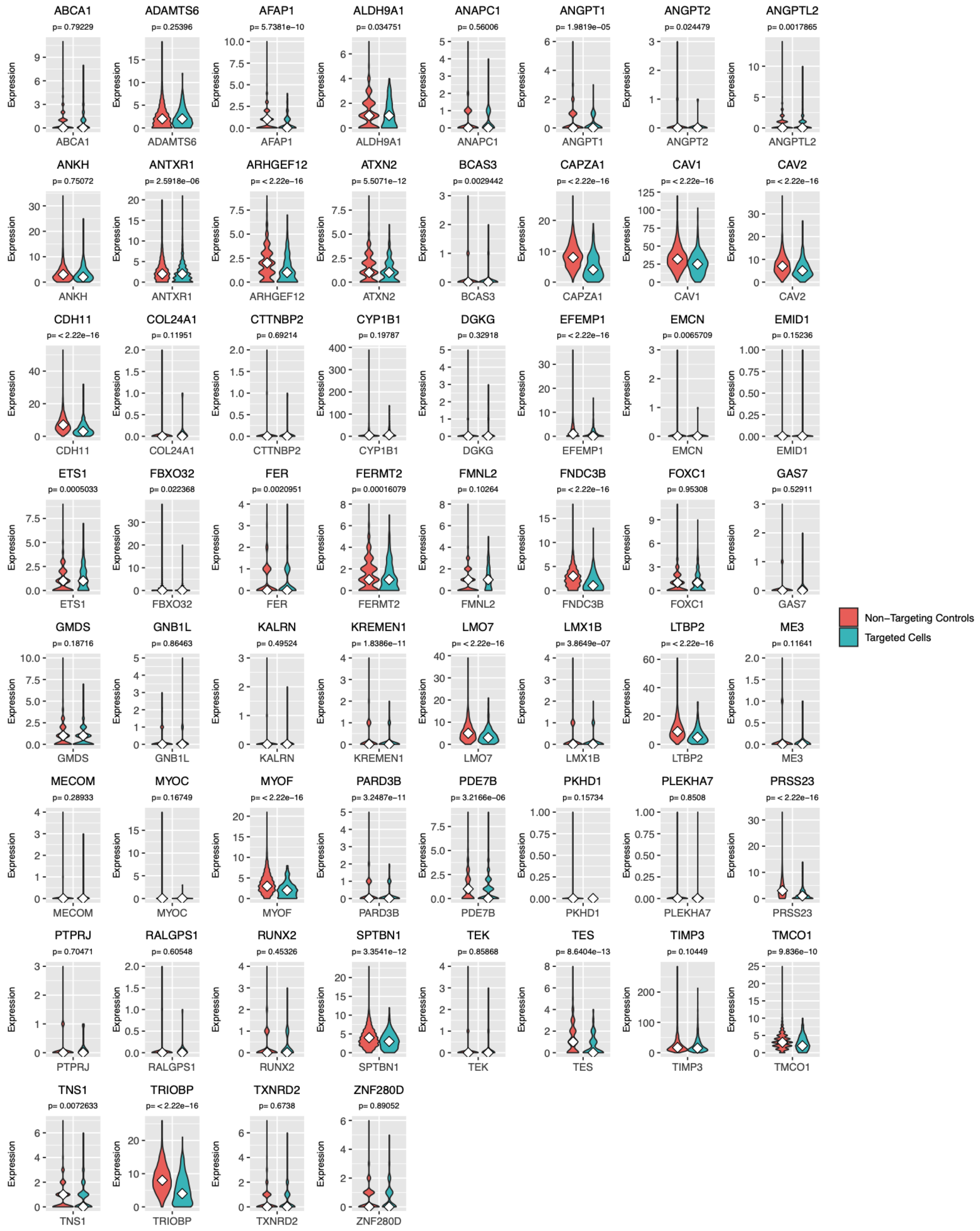

Supplement: Fig S2 [file mmc3.pdf]
