# Supplementary material for: Deep Learning-Based Identification of Intraocular Pressure-Associated Genes Influencing Trabecular Meshwork Cell Morphology
Source: Ophthalmol Sci. 2024 Mar 5;4(4):100504. doi: 10.1016/j.xops.2024.100504 (PMC11046128; doi:10.1016/j.xops.2024.100504)
Supplement: Table S1 [file mmc4.pdf]

Supplementary table 1

sgRNA name	Sequence	Gene	Exon	Protein domain targeted	Predicted On-target efficiency
GUIDES_sg001	GTTGACTGGGAGAGAACACG	*ABCA1*	46	ABC_tran	0.707186352
GUIDES_sg002	GTGTTCTAAAAGAGAAACAC	*ABCA1*	50	-	0.684107302
GUIDES_sg003	GTGCAGTGTCTCTCCTACAC	*ADAMTS6*	28	-	0.720054847
GUIDES_sg004	ACCAGTCATGTCCACCACAG	*ADAMTS6*	26	-	0.71972146
GUIDES_sg005	CAAGGGTAAAAAGCCCCCCG	*AFAP1*	16	-	0.759587174
GUIDES_sg006	GGAAAGAAAAGACCTTCGAG	*AFAP1*	17	-	0.753862205
GUIDES_sg007	CAACTATAACGTCAGCCCAG	*ALDH9A1*	10	Aldedh	0.764185557
GUIDES_sg008	TATGAACAATGCTGTAAAGG	*ALDH9A1*	6	Aldedh	0.704113168
GUIDES_sg009	GTGCTGTGAGCTGGGAAGTG	*ANAPC1*	39	-	0.69840945
GUIDES_sg010	ATGGCTCTTCCTGTAGGACG	*ANAPC1*	27	-	0.641611628
GUIDES_sg011	CCCCAGCCAATATTCACCGG	*ANGPT1*	9	Fibrinogen_C	0.666179972
GUIDES_sg012	AATATGGATGTCAATGGGGG	*ANGPT1*	8	Fibrinogen_C	0.630390739
GUIDES_sg013	TAACGTGTAGATGCCATTCG	*ANGPT2*	5	Fibrinogen_C	0.692502215
GUIDES_sg014	TGTGACATGGAAGCTGGAGG	*ANGPT2*	6	Fibrinogen_C	0.647042655
GUIDES_sg015	ACTCGCTCTCAGGTTCCAGG	*ANGPTL2*	5	Fibrinogen_C	0.761119961
GUIDES_sg016	CACCAGCATGTCACGCACAG	*ANGPTL2*	2	RasGEF	0.753686491
GUIDES_sg017	CTTTGTGGGAGAATCCACCA	*ANKH*	14	ANKH	0.705623542
GUIDES_sg018	TGAGGGCGCATCTCACCGGG	*ANKH*	13	ANKH	0.671178514
GUIDES_sg019	CTTCCGACATGCCCGCAACG	*ANTXR1*	10	Anth_Ig	0.66965417
GUIDES_sg020	CAGAACTGGAGATAAAAGAG	*ANTXR1*	12	Anth_Ig	0.661995855
GUIDES_sg021	CTGCTGGACCAGAAATTCGG	*ARHGEF12*	39	-	0.715629912
GUIDES_sg022	TCTCTGGGGTCATAATCATG	*ARHGEF12*	38	-	0.682338139
GUIDES_sg023	TACCAAATATGCCCCAACAG	*ATXN2*	21	-	0.746394842
GUIDES_sg024	ATTACAGGACTATAGACATG	*ATXN2*	22	-	0.705381212
GUIDES_sg025	ATGGGCCCAGGACTTCCAGG	*BCAS3*	35	-	0.763805308
GUIDES_sg026	TGAACTGGATGAGATAACTG	*BCAS3*	36	-	0.749363581
GUIDES_sg027	AGTTTTTAGGCTGAAACTGG	*CAPZA1*	6	F-actin_cap_A	0.671121892
GUIDES_sg028	GGAATAATGGTCTTTCACAT	*CAPZA1*	5	F-actin_cap_A	0.61837895
GUIDES_sg029	TAAACACCTCAACGATGACG	*CAV1*	3	Caveolin	0.697766064
GUIDES_sg030	TGGGGGCAAATACGTAGACT	*CAV1*	1	Caveolin	0.638532867
GUIDES_sg031	GATGTGCAGACAGCTGAGGG	*CAV2*	23	Caveolin	0.699222046
GUIDES_sg032	CGGCGTACTCGAGGCCGCTG	*CAV2*	22	Caveolin	0.674769146
GUIDES_sg033	TCAGGAAGCCAAAGTCCCAG	*CDH11*	19	Cadherin	0.765145054
GUIDES_sg034	GGATTGTGAATGATTTCAGG	*CDH11*	20	Cadherin	0.686839641
GUIDES_sg035	GGGTTATCTCGTGTGCCAAG	*COL24A1*	60	COLFI	0.662833491
GUIDES_sg036	GAAATTGCAGAAAACCTCAA	*COL24A1*	61	COLFI	0.614109727
GUIDES_sg037	AAGCGGCCAGACTTCCTGCG	*CTTNBP2*	25	-	0.737788504
GUIDES_sg038	GCCAGGTTGTCTTTTCACAG	*CTTNBP2*	24	-	0.682279464
GUIDES_sg039	AACATTCCCAGCATGTACGG	*DGKG*	22	DAGK_acc	0.746132205
GUIDES_sg040	GTACTTTGAATTTGGCACCT	*DGKG*	21	DAGK_acc	0.610555363
GUIDES_sg041	GACCACAAATGAATGCCGGG	*EFEMP1*	9	EGF_CA	0.7383367

GUIDES_sg042	TCACCACTTGGTATCCCTGG	*EFEMP1*	8	EGF_CA	0.711733541
GUIDES_sg043	ACGCTCTCTTTATCAGACTG	*EMCN*	19	Endomucin	0.716120299
GUIDES_sg044	GTTTTAGAAGGTGATGCATC	*EMCN*	15	Endomucin	0.500046723
GUIDES_sg045	AGGACTCCCAGGGACACCTG	*EMID1*	11	Collagen	0.633805274
GUIDES_sg046	GCTGCCCAGCAGAGCCTTGG	*EMID1*	13	Collagen	0.50154336
GUIDES_sg047	GCAGTGGACCAATCCAGCTA	*ETS1*	13	Ets	0.590477455
GUIDES_sg048	CACTAAAGAACAGCAACGAC	*ETS1*	8	SAM_PNT	0.507782957
GUIDES_sg049	ATGTTCAGTTGTAGGCACAA	*FBXO32*	7	-	0.680491123
GUIDES_sg050	AACTTGTCCGATGTTACCCA	*FBXO32*	8	-	0.665922839
GUIDES_sg051	ACAGTTCAGAGAGTGACGTG	*FER*	26	Pkinase	0.744231001
GUIDES_sg052	ATGTCTCGTCAAGAGGATGG	*FER*	25	Pkinase	0.666176654
GUIDES_sg053	GCCTGCAGATTAGCCTCCAA	*FERMT2*	17	PH	0.635135622
GUIDES_sg054	CTGAGGTTCATCTGATGAGC	*FERMT2*	15	PH	0.496718402
GUIDES_sg055	GGAGTTCATCCTCAACAATG	*FMNL2*	23	FH2	0.656016612
GUIDES_sg056	TTCACAAACCGGACAAAGAC	*FMNL2*	24	FH2	0.52306371
GUIDES_sg057	TGTGTACACACTACAGCTGG	*FNDC3B*	31	fn3	0.726749897
GUIDES_sg058	GCTCTTCCCAGTTCAGTACA	*FNDC3B*	30	fn3	0.69274705
GUIDES_sg059	GCTGAGCAACAAGACAGAGG	*GAS7*	19	-	0.715444147
GUIDES_sg060	GCTTGCGAAGGTCGGCAATG	*GAS7*	18	-	0.692490099
GUIDES_sg061	GCCCACACTCTCCAAGCACA	*GNB1L*	3	-	0.656080017
GUIDES_sg062	GCAGGCTCCAGATGTGTACC	*GNB1L*	2	WD40	0.572759585
GUIDES_sg063	TGAACAGAGAGACTTCTGAG	*KALRN*	59	PH	0.713929927
GUIDES_sg064	CTTCCTGAGATACAGTGAGA	*KALRN*	56	RhoGEF	0.590046749
GUIDES_sg065	TGATTACTGGAAGTACGGGG	*KREMEN1*	5	WSC	0.732200895
GUIDES_sg066	TTACTGGTGCCAGTTAGAGG	*KREMEN1*	4	WSC	0.660612633
GUIDES_sg067	CAGGGACTCGATGATCATGG	*LMO7*	34	LIM	0.767439497
GUIDES_sg068	GATCCTGACTTCAGCTCCTG	*LMO7*	35	LIM	0.666458078
GUIDES_sg069	CTTCGACGAGACCTCGAAGG	*LMX1B*	4	Homeobox	0.713403988
GUIDES_sg070	GTGCAAGGGTGACTACGAGA	*LMX1B*	3	LIM	0.675803927
GUIDES_sg071	ACGTCTCGGATGGTGCTGAG	*ME3*	18	Malic_M	0.711900963
GUIDES_sg072	AGAGAAAGAAGGTGTACCGA	*ME3*	14	Malic_M	0.683948993
GUIDES_sg073	TAGTACTTCCCATGTGCCAG	*MECOM*	24	-	0.69054325
GUIDES_sg074	ACTGTGGCAAGATTTTTCCA	*MECOM*	20	zf-C2H2	0.638498746
GUIDES_sg075	GGACTTCTGCTCAAAGAGGG	*MYOF*	56	-	0.689398449
GUIDES_sg076	TGCATGGGTTGGTGAACCAG	*MYOF*	58	-	0.68114604
GUIDES_sg077	AAAGGTACTCTGAAACATGG	*PARD3B*	24	-	0.748915706
GUIDES_sg078	TGGTCTCTTTCTGGAGACAG	*PARD3B*	25	-	0.687131379
GUIDES_sg079	TCTGGGAGATGAGCAAGCAG	*PDE7B*	11	PDEase_I	0.63710316
GUIDES_sg080	TCTTTCTGTTGATTACAAAG	*PDE7B*	12	PDEase_I	0.626787508
GUIDES_sg081	CGTGAGACTCCAGTCACAGG	*PKHD1*	20	-	0.721146822
GUIDES_sg082	ATGGGATAGCCCCAAGCAGG	*PKHD1*	16	-	0.648027027
GUIDES_sg083	TACTCAGGGGATCACCAGCG	*PLEKHA7*	28	-	0.733493753
GUIDES_sg084	CCCCGAACTCTACAGCCCAG	*PLEKHA7*	25	-	0.727012157
GUIDES_sg085	GCTGCCCACTGCATACACGA	*PRSS23*	3	Trypsin	0.707785666

GUIDES_sg086	AACATCAGTGAAGTTATCCA	*PRSS23*	3	Trypsin	0.589826381
GUIDES_sg087	CAGTGGTGTCGGGAACACCG	*PTPRJ*	26	Y_phosphatase	0.752294442
GUIDES_sg088	GTTCGGTAAAGGTCCTTGTG	*PTPRJ*	24	Y_phosphatase	0.742428198
GUIDES_sg089	TGGCAAAAAGGTTTCCATCG	*RALGPS1*	25	PH	0.63983389
GUIDES_sg090	CGAAAGAAGATAATTACAAG	*RALGPS1*	11	RasGEF	0.632295378
GUIDES_sg091	AGAGGTACCAGATGGGACTG	*RUNX2*	5	Runt	0.707376125
GUIDES_sg092	CATGGCGGAAGCATTCTGGA	*RUNX2*	11	RunxI	0.681198759
GUIDES_sg093	TGGAATTCCCTACCACAGCG	*SPTBN1*	36	PH	0.712246448
GUIDES_sg094	TCAGTCTTAACCATTCCCAT	*SPTBN1*	31	Spectrin	0.683179693
GUIDES_sg095	GGGCTGGCTATGATAAACTG	*TES*	6	LIM	0.774116567
GUIDES_sg096	CCATGAGTTGTCTCCCAGAG	*TES*	5	PET	0.735126362
GUIDES_sg097	GAAGCTTCCGAGAGTCTCTG	*TIMP3*	3	TIMP	0.703685264
GUIDES_sg098	CTATGATGGCAAGATGTACA	*TIMP3*	4	TIMP	0.591387438
GUIDES_sg099	AGTCCTTGGATGTAAGAAAG	*TMCO1*	9	DUF841	0.652850785
GUIDES_sg100	GAAACAATAACAGAGTCAGC	*TMCO1*	5	DUF841	0.622258097
GUIDES_sg101	AGAGACTTTGAAGTGAACGA	*TNS1*	42	PTB	0.715095311
GUIDES_sg102	CAGAAGGTGACAGTGTTGAG	*TNS1*	43	PTB	0.675343539
GUIDES_sg103	GCCGACTGGTGACCTCATGG	*TRIOBP*	3	-	0.713915639
GUIDES_sg104	GGGAGCAGGAGGCAGGAACG	*TRIOBP*	4	-	0.656603289
GUIDES_sg105	TAAACCACTGGAGTTCACGG	*TXNRD2*	20	Pyr_redox_dim	0.785275668
GUIDES_sg106	TCATCATTGCTACTGGAGGG	*TXNRD2*	8	Pyr_redox_2	0.706088692
GUIDES_sg107	GGTGAAGCTCCTGATTGCAG	*ZNF280D*	27	-	0.700021186
GUIDES_sg108	GAAGAAAGTAAAAGAAGTTG	*ZNF280D*	15	-	0.599965492
GUIDES_sg109	ATGGAGTTCCGCGACCACGT	*ABO*	7	CDS	0.6563
GUIDES_sg110	CCGGTCCCCAGCGTCACGCG	*ABO*	7	CDS	0.6687
GUIDES_sg111	CCACCTGGTACATCGCCTCA	*TEX41*	2	TRANSCRIPT	0.6631
GUIDES_sg112	AACTCAAGACATTGGAACCA	*TEX41*	5	TRANSCRIPT	0.6251
GUIDES_sg113	AATGTGGTAGCCCAAGACAG	*CYP1B1*	5	p450	0.775320729
GUIDES_sg114	GTGGCCACTGATCGGAAACG	*CYP1B1*	3	p450	0.726256031
GUIDES_sg115	GCAAGCCATGAGCCTGTACG	*FOXC1*	1	-	0.747984594
GUIDES_sg116	TCGTCGTCCCTGAGTCACGG	*FOXC1*	1	-	0.730741126
GUIDES_sg117	GATTGTGGTGAACTTCCGTG	*GMDS*	8	Epimerase	0.727535334
GUIDES_sg118	GTTGCAGAATGATGAGCCGG	*GMDS*	10	Epimerase	0.65599947
GUIDES_sg119	CCTCCCGCACGCGCACACAG	*LTBP2*	35	EGF	0.755863507
GUIDES_sg120	CAGGCAGACATAACCAGGCA	*LTBP2*	31	EGF_CA	0.708717
GUIDES_sg121	GGTCATACTCAAAAACCTGG	*MYOC*	3	OLF	0.763937898
GUIDES_sg122	ATGCCAGTATACCTTCAGTG	*MYOC*	1	-	0.722928246
GUIDES_sg123	TCTTGCGAAGGAAGTCCAGA	*TEK*	17	Pkinase	0.627785812
GUIDES_sg124	ATCTAATGAGACAATGCTGG	*TEK*	22	Pkinase	0.626682424
NonTargeting Human_0001	ACGGAGGCTAAGCGTCGCAA	-	-	-	-
NonTargeting Human_0002	CGCTTCCGCGGCCCGTTCAA	-	-	-	-
NonTargeting Human_0003	ATCGTTTCCGCTTAACGGCG	-	-	-	-

NonTargeting Human_0004	GTAGGCGCGCCGCTCTCTAC	-	-	-	-
NonTargeting Human_0005	CCATATCGGGGCGAGACATG	-	-	-	-
NonTargeting Human_0006	TACTAACGCCGCTCCTACAG	-	-	-	-
NonTargeting Human_0007	TGAGGATCATGTCGAGCGCC	-	-	-	-
NonTargeting Human_0008	GGGCCCGCATAGGATATCGC	-	-	-	-
NonTargeting Human_0009	TAGACAACCGCGGAGAATGC	-	-	-	-
NonTargeting Human_0010	ACGGGCGGCTATCGCTGACT				
