# Supplementary material for: Deep Learning-Based Identification of Intraocular Pressure-Associated Genes Influencing Trabecular Meshwork Cell Morphology
Source: Ophthalmol Sci. 2024 Mar 5;4(4):100504. doi: 10.1016/j.xops.2024.100504 (PMC11046128; doi:10.1016/j.xops.2024.100504)
Supplement: Table S2 [file mmc5.pdf]

Supplementary table 2 - Tensorflow CNN architecture

Layer	Output shape	Trainable parameters
conv2d (Conv2D)	(None, 54, 54, 96)	34,944
activation (relu)	(None, 54, 54, 96)	0
max_pooling2d (MaxPooling2D)	(None, 27, 27, 96)	0
conv2d_1 (Conv2D)	(None, 17, 17, 256)	2,973,952
activation_1 (relu)	(None, 17, 17, 256)	0
max_pooling2d_1 (MaxPooling2D)	(None, 8, 8, 256)	0
conv2d_2 (Conv2D)	(None, 6, 6, 384)	885,120
activation_2 (relu)	(None, 6, 6, 384)	0
conv2d_3 (Conv2D)	(None, 4, 4, 384)	1,327,488
activation_3 (relu)	(None, 4, 4, 384)	0
conv2d_4 (Conv2D)	(None, 2, 2, 256)	884,992
activation_4 (relu)	(None, 2, 2, 256)	0
max_pooling2d_2 (MaxPooling2D)	(None, 1, 1, 256)	0
flatten (Flatten)	(None, 256)	0
dense (Dense)	(None, 4096)	1,052,672
activation_5 (relu)	(None, 4096)	0
dropout (Dropout)	(None, 4096)	0
dense_1 (Dense)	(None, 4096)	16,781,312
activation_6 (relu)	(None, 4096)	0
dropout_1 (Dropout)	(None, 4096)	0
dense_2 (Dense)	(None, 1000)	4,097,000
activation_7 (relu)	(None, 1000)	0
dropout_2 (Dropout)	(None, 1000)	0
dense_3 (Dense)	(None, 2)	2,002
activation_8 (softmax)	(None, 2)	0
Total trainable parameters		28,039,482
